# Detection of Hepatitis E Virus (HEV) in Pork Sold in Saint-Louis, the North of Senegal

**DOI:** 10.3390/life14040512

**Published:** 2024-04-16

**Authors:** Sophie Deli Tene, Abou Abdallah Malick Diouara, Alé Kane, Sarbanding Sané, Seynabou Coundoul, Fatou Thiam, Cheikh Momar Nguer, Mamadou Diop, Mame Ndew Mbaye, Malick Mbengue, Seynabou Lo, Halimatou Diop Ndiaye, Coumba Toure Kane, Ahidjo Ayouba

**Affiliations:** 1 Groupe de Recherche Biotechnologies Appliquées & Bioprocédés Environnementaux (GRBA-BE), École Supérieure Polytechnique (ESP), Université Cheikh Anta Diop, Dakar 5085, Senegal; sophiedelitene@esp.sn (S.D.T.); sarbandingsane@esp.sn (S.S.); seynaboucoundoul@esp.sn (S.C.); fatou54.thiam@ucad.edu.sn (F.T.); cmnguer@gmail.com (C.M.N.); mamadou.diop1@esp.sn (M.D.); mamendew.mbaye@esp.sn (M.N.M.); 2 Laboratoire des Sciences Biologiques, Agronomiques, Alimentaires et de Modélisation des Systèmes Complexes (LABAAM), UFR S2ATA, Université Gaston Berger, Saint-Louis 234, Senegal; ale.kane@ugb.edu.sn; 3 Laboratoire de Microbiologie Appliquée et de Génie Industriel, École Supérieure Polytechnique (ESP), Université Cheikh Anta Diop, Dakar 5085, Senegal; malick.mbengue@esp.sn; 4 Unité de Formation et de Recherche Science de la Santé (UFR 2S), Université Gaston Berger, Saint-Louis 234, Senegal; seynabou.lo@ugb.edu.sn; 5 Laboratoire de Bactériologie Virologie CHU Aristide le Dantec, Université Cheikh Anta DIOP, Dakar 5005, Senegal; halimatoudiop@yahoo.fr; 6 Institut de Recherche en Santé, de Surveillance Épidémiologique et de Formation (IRESSEF), Pole Urbain Diamniadio, Dakar 7325, Senegal; coumba.toure@iressef.org; 7 Université Sine Saloum El Hadj Ibrahima Niass (USSEIN), Kaolack 55, Senegal; 8 Recherches Translationnelles sur le VIH et Maladies Infectieuses, Institut de Recherche pour le Développement, Université de Montpellier/INSERM U1175, 34000 Montpellier, France; ahidjo.ayouba@ird.fr

**Keywords:** hepatitis E virus, HEV-3, foodborne disease, domestic wildlife, zoonotic risk, Saint-Louis

## Abstract

The hepatitis E virus (HEV) is a zoonotic pathogen with various hosts, including pigs, which act as reservoirs. In industrialized countries, sporadic cases caused by genotype 3, contracted by ingesting contaminated uncooked or undercooked meat, have been reported. However, in developing countries, HEV infection is mainly dominated by genotype 2 and often associated with poor hygiene conditions and drinking water supplies. HEV infection and its circulation in domestic fauna in West Africa are poorly documented. This study aimed to assess the presence of HEV in pork sold in Saint-Louis, Senegal. Meat products (250 g samples, *n* = 74) were purchased in August 2022 from three locations. Then, 2 g/sample was minced to extract total nucleic acids using the Purelink™ Viral DNA/RNA kit. RT-PCR reactions were performed using the One-Taq™ One-Step RT-PCR kit targeting the HEV ORF2 genomic region. The products obtained were visualized on a 1% agarose gel. Of a total of 74 samples, divided into pork meat (*n* = 65) and pork liver (*n* = 9), 5.4% (*n* = 4) tested positive for HEV. In both cases, two samples were positive, representing a rate of 3.1% and 22.2% for meat and pork liver, respectively. All new viral sequences were obtained from a monophyletic group within HEV genotype 3. This study is the first to report the presence of HEV in pork sold in Senegal and the results reveal a potential circulation of HEV in the pig population. The high proportion of contamination in the pork liver samples highlights a major risk associated with their consumption.

## 1. Introduction

Hepatitis E virus (HEV) is a small enveloped or quasi-enveloped virus with an icosahedral capsid. Its genome is a single-stranded positive sense monopartite RNA with a ~6.4–7.2 bp size, expressing three open reading frames (ORFs): ORF1, ORF2, and ORF3 [1,2,3]. According to a 2021 release by the International Committee on the Taxonomy of Viruses (ICTV), HEV belongs to the *Hepeviridae* family, which is divided into two subfamilies: *Orthohepevirinae* (detected in mammals and birds) and *Parahepevirinae* (only detected in fish). HEV is classified in eight different genotypes (HEV-1 to HEV-8), all belonging to *Paslahepevirus balayani* and infecting humans and numerous domestic and wild mammals [2,3]. To date, four main HEV genotypes 1–4 are known to infect humans [4]. Furthermore, numerous studies show that most HEV infections in pigs are caused by HEV-3, which is widely spread across the globe, while HEV-4 is less prevalent in the global pig population [5]. Now, HEV is recognized as a biological hazard that must be controlled and is an etiological agent in viral hepatitis [6]. From a genetic point of view, HEV genotypes 3 and 4 are distinguished by greater diversity. In their well conducted review paper on the hepatitis E in livestock, Turlewicz-Podbielska et al. point out the phylogenetic relationships showing a total of three main clades of HEV-3: clade 3.1, which includes subtypes a, b, c, h, i, and j; clade 3.2, which includes subtypes e, f, and g; and clade 3.3, which contains rabbit strains corresponding to the HEV-3ra subtype. Nine subtypes were determined among HEV-4: 4a–4i [5].

According to the World Health Organization (WHO), HEV is now considered as an emerging public health concern worldwide, causing large outbreaks and sporadic cases of acute hepatitis [7]. It causes an estimated 20 million infections per year, leading to 3.3 million symptomatic cases and 44,000 deaths, accounting for 3.3% of mortality due to viral hepatitis. HEV infection is one of the leading causes of acute viral hepatitis among adults in Asia, the Middle East, and Africa [8,9]. Most infections are self-limited acute hepatitis in immunocompetent subjects and asymptomatic cases are often found; in the general population, the mortality rate ranges from 0.5 to 4% [10,11,12,13]. However, immunosuppressed subjects, pregnant women, and individuals with preexisting chronic liver disease are considered as a risk group for hepatitis E infection. These vulnerable populations may experience a more severe course of infection and have a poorer prognosis [8,10,14,15,16,17]. In pregnant women infected with HEV, the mortality rate rises up to 30% because they are more likely to develop complicated forms of the disease [10,18,19,20,21].

HEV is mainly transmitted via fecal–oral routes, blood transfusion, the environment through contaminated crops, and exposure to wastewater from toilets or via floods in the rainy season [22]. Genotypes 1 and 2 infecting only humans are transmitted through fecal–oral routes and are responsible for large HEV outbreaks in resource-limited countries. These have mainly occurred via fecal contamination of drinking water due to poor water supplies and lack of hygiene and sanitation [23]. There is considerable epidemiological evidence of waterborne HEV transmission, especially in Southern/South-East/Central Asia and North West Africa [24,25,26]. In contrast, genotypes 3 and 4, circulating primarily among mammalian animals, are responsible for zoonotic transmission [4,27,28,29]. Pigs are omnivorous; hence, like humans, they are frequently infected via the fecal–oral route. Pigs could be contaminated through the consumption of contaminated food. Also, they may act as reservoirs of HEV-1 through an inter-species transmission mechanism [5,30]. The remaining genotypes (HEV-2 and HEV-5 to HEV-8) have not been documented in pigs yet [5].

In industrialized countries and in areas with good sanitation and water supplies, only sporadic cases occur caused by genotype 3 acquired through zoonotic HEV infection by ingestion of contaminated foodstuffs and particularly by eating undercooked contaminated meat (pig flesh, raw liver, sausages, etc.) [22,31]. However, in developing countries, large outbreaks of HEV-1 and HEV-2 have manifested with increasing frequency [32]. Consistently, most studies have reported poor water supplies and sanitation as the main associated risk factors for HEV infection [6,10,33]. HEV shed by infected animals can contaminate water sources and lead to the accumulation of viruses in the environment. Infectious HEV has also been found in swine manure and wastewater [34]; therefore, application of manure as a soil fertilizer and subsequent runoff could contaminate cultivations and coastal water, leading to contamination of crops and shellfish and, subsequently, possible human infection. The feces are the biological matrix with the highest probability of detecting HEV [35]. Furthermore, HEV-3 RNA has also been detected in red fruit, strawberries, salad greens, and spices [36], as well as in oysters and mussels [37].

HEV is a zoonotic pathogen which has a large host range, being found in humans as well as in a wide range of domestic and wild mammals (pig, wild boar, cow, deer, rabbit, camel) [2]. The expanded host range indicates the high variability of these HEV strains and their zoonotic potential [38,39]. Autochthonous HEV infections have been reported in many developed countries by contact with infected animals [40]. The domestic pig (*Sus scrofa domesticus*) is an important reservoir host of HEV and is a source of contamination for the consumer after consuming raw or undercooked pork products [41,42]. The liver is the target organ for HEV and is where it replicates, but it was also detected in several other tissues [43]. Infected pigs are usually without any apparent clinical symptoms, although in some cases, mild to moderate, acute, self-limiting hepatitis can occur [43,44]. Consequently, HEV-infected pigs enter the slaughterhouse as healthy animals; hence, their tissues and meat can pose a risk to human consumers. Pork liver and its derivatives are more frequently found to be positive for HEV-RNA, and therefore, are the most obvious sources of foodborne HEV [43,45,46,47]. Consumption of these products underlines direct evidence of zoonotic HEV transmission. Many studies reported the detection of HEV in 1.3%, 1.9%, 11%, and 6.5% of pig livers sold in retail stores in England, Japan, the USA, and The Netherlands, respectively [48,49,50,51]. On the other hand, several reports describe human cases of Hepatitis E associated with foodborne transmission involving products containing undercooked raw pork liver [50,52,53,54]. Furthermore, the virus has also been found in muscle tissue, which could be a source of human infection [55].

The first documented outbreak of HEV infection in Africa likely occurred in 1950 in Tunisia [56]. In a systematic review of African studies, at least 17 outbreaks were reported in Africa about once every other year since 1979 in 28 countries out of 56, causing a reported 35,300 cases with 650 deaths [10,57]. The most recent epidemics reported in Africa occurred in 2017 (Niger, Nigeria, and Chad) and 2018 (Namibia) [58]. On the other hand, a systematic review was conducted on the prevalence of HEV in animals in Africa, highlighting that some animals, such as pigs, could be the reservoir of HEV genotype 3 (HEV-3) and suggesting the need for molecular epidemiological studies for investigating zoonotic transmission in Africa [22,59]. Additionally, genotype 3 has been isolated in Cameroon in domestic pigs, and this genotype seems to have an extensive distribution that includes Africa [60]. An HEV genotype 3 strain was also identified in domestic pigs from Kinshasa, the Democratic Republic of the Congo, suggesting an importation from Belgium to Congo through animal trading [61].

Indeed, in African countries, few studies have focused on HEV as a zoonotic and a foodborne pathogen. Furthermore, HEV problematic in foodstuffs, mainly in domestic fauna, is poorly covered in West Africa, particularly in Senegal, where we note a lack of data related to the national prevalence of HEV as well as epidemiological data reporting the circulating genotypes in this area. In view of all the above-mentioned aspects, it has become necessary to elucidate the possible sources of zoonotic HEV contamination in Senegal in order to understand the risks associated with the consumption of pork products. This is the first study to investigate the presence of HEV in pork meat and liver sold in retail in Saint-Louis, Senegal.

## 2. Materials and Methods

### 2.1. Sampling

**Figure 1:**
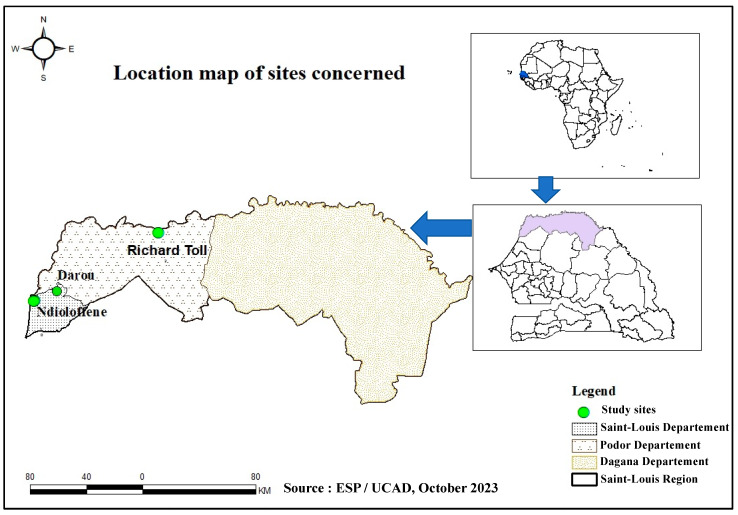
Sampling site locations in Saint-Louis.

This one-site study is part of an HEV molecular surveillance program in Senegal using a One Health approach. It represents phase II of a pilot study conducted by Diouara et al., focusing on the HEV seroprevalence and associated risk factors in pregnant women in Senegal [10]. In this present study, samples of pork meat and liver were collected between 20 and 25 August 2022 in three locations in Saint-Louis, namely Ndioloffène, Darou, and Richard-Toll (Figure 1). Unlike other meat products, pork is often sold there in houses or bars. The latter is well known to consumers, so these sites were chosen on the basis of their reputation, their capacity to receive consumers daily, and pork availability during our sampling period.

Samples were obtained by purchasing approximately 250 g of pork meat and/or liver, with a minimum of three and a maximum of five samples per vendor to ensure good representativeness and in accordance with the recommendations for sampling food matrices [62]. A unique code was assigned to each sample, which was placed in an individual sterile zipped bag and maintained in an isothermal transport bag between 4–8 °C in order to preserve the integrity of products in accordance with validated procedures [10,63]. Once in the laboratory, the samples were stored in the GRBA-BE biobank at −20 °C until their use.

### 2.2. Extraction of Total Nucleic Acid (TNA)

For extraction needs, the raw meat products were taken from −20 °C. After defrosting at room temperature, we mince the corresponding sample, ensuring that the mass is representative of the sample in question. In other words, we touch each piece of meat in the sample to ensure a homogeneous mince. We then weighed 2 g of this minced meat and suspended it in sterile physiological water (0.9% NaCl) to prepare the mother suspension. Subsequently, the suspension is vigorously vortexed for up to 10 min to obtain a highly homogeneous paste or broth. These steps were followed by centrifugation at 3000 rpm for 10 min to remove tissue debris. A volume of 200 µL of the supernatant containing cells and any viral particles was subjected to hot lysis with the corresponding buffer as recommended. TNA was extracted with the Purelink™ Viral DNA/RNA Mini kit (PureLink) (Thermo Fisher Scientific, Waltham, MA, USA) according to the manufacturer’s instructions. The elution volume was 50 μL, and TNA samples were stored at −80 °C until further analysis.

### 2.3. RT-PCR

For HEV detection, we targeted the ORF2 genomic region with the specific primer pair HE040 (5′-CCCTTRTCCTGCTGAGCRTTCTC-3′ [R = A or G]) and HE044 (5′-CAAGGHTGGCGYTCKGTTGAGAC-3′ [H = A, T or C; Y = T or C; and K = G or T]) as described by Mizuo et al. [64]. One-step RT-PCR was performed using the One-Taq^®^ One-Step RT-PCR kit (New England Biolabs, MA, USA) with the following thermocycling program: 48 °C for 25 min for the reverse transcription step; an initial denaturation step of 2 min at 94 °C followed by 40 cycles of 94 °C for 15 s; 55 °C for 30 s and 68 °C for 1 min, and a final extension step of 5 min at 68 °C. In this study, we used a confirmed HEV-positive sample from our laboratory as a positive control. For the negative control, we used high molecular quality water, i.e., the nuclease-free water provided in the kit used. The amplicons obtained were then visualized via electrophoresis on a 1% agarose gel with an expected size of 506 bp. The manipulation was validated when the positive control showed a band at the expected size and when the negative control showed no band.

### 2.4. Sequencing and Phylogenetic Analysis

Oxford Nanopore Technologies (ONT) sequencing libraries were prepared from ORF2 genomic region amplicon using the Rapid barcoding sequencing (SQK-RBK110.96) kit according to the manufacturer’s protocol; sequencing was undertaken on the MinIon MK1C device with R9.4.1 (FLO-MIN 106) flowcells for 5 h. A minimal Qscore of 7 was considered. In the end, the raw fastQ files were recovered for sequence analysis.

Firstly, the fastQ files for each barcode were converted into fasta files using online software (https://sequenceconversion.bugaco.com/converter/biology/sequences/fastq_to_fasta.php, accessed on 6 December 2023). Next, the sequences corresponding to HEV were extracted from the fasta files with R software (version 4.2.3) for each of the barcodes via the sequence IDs retrieved through the Epi2me csv report.

For the phylogenetic analysis, partial sequences of HEV ORF-2 capsid (starting at position 5895 and ending at position 6382 relative to NC_001434 reference sequence) were subject to multiple sequence alignment with MUSCLE and gap positions were removed using the Gblocks program on SEAVIEW software v5.0.4 [65]. A set of HEV reference sequences downloaded from GenBank was used for this purpose. Phylogenetic trees were inferred using the Maximum Likelihood method with PhyML v3.1 [66]. The tree was generated under the best-fit nucleotide substitution model GTR + G + I determined using MODELTEST in MEGA X [67]. The Subtree–Pruning–Regrafting heuristic search was applied for optimal tree topology. Branch supports were determined by the approximate likelihood ratio test method (aLRT) SH-like option [68]. The phylogenetic tree was read and edited with Figtree [69].

### 2.5. Statistical Analysis

Data obtained were analyzed using Excel and R version 4.2.3 (15 March 2023) with default settings. Positivity rates of HEV-RNA were calculated, and 95% confidence intervals were obtained using proportionality tests. In order to measure possible associations, Fisher’s exact test was performed, and a difference was considered statistically significant when the *p*-value was less than 0.05.

## 3. Results

A total of 74 samples were collected at Saint-Louis in the following distribution: 65 pork meat samples and nine pork liver samples. The Table 1 shows the distribution of samples by collection site and by sample type. All pig liver samples (*n* = 9) collected in this study came from Ndioloffène. As for the pork meat samples, *n* = 29, *n* = 17 and *n* = 19 were collected at Ndioloffène, Richard Toll, and Darou, respectively (Table 1).

The RT-PCR results showed an overall HEV-RNA prevalence of 5.4% (*n* = 4); 95% CI [1.7–14%] in pork products. It should be noted that all HEV-RNA positive samples were collected at Ndioloffène, i.e., a positivity rate of 10.5% (*n* = 4); 95% CI [3.4–25.7%] when considering the only site. No HEV positivity was found in samples collected at Darou and Richard-Toll (Table 1). Statistical analysis reveals no significant difference between the collection site and the prevalence of HEV in pork products screened (*p* = 0.1698).

According to the sample type, pig meat and liver samples show 3.1%; 95% CI [0.53–11.6%] and 22.2%; 95% CI [3.9–59.8%] of HEV-RNA positivity, respectively. In our study, while a higher HEV-RNA positivity rate was found in pork liver samples, no statistically significant association was found with the types of samples (meat or liver) analyzed (*p* = 0.0699) (Table 2).

**Figure 2:**
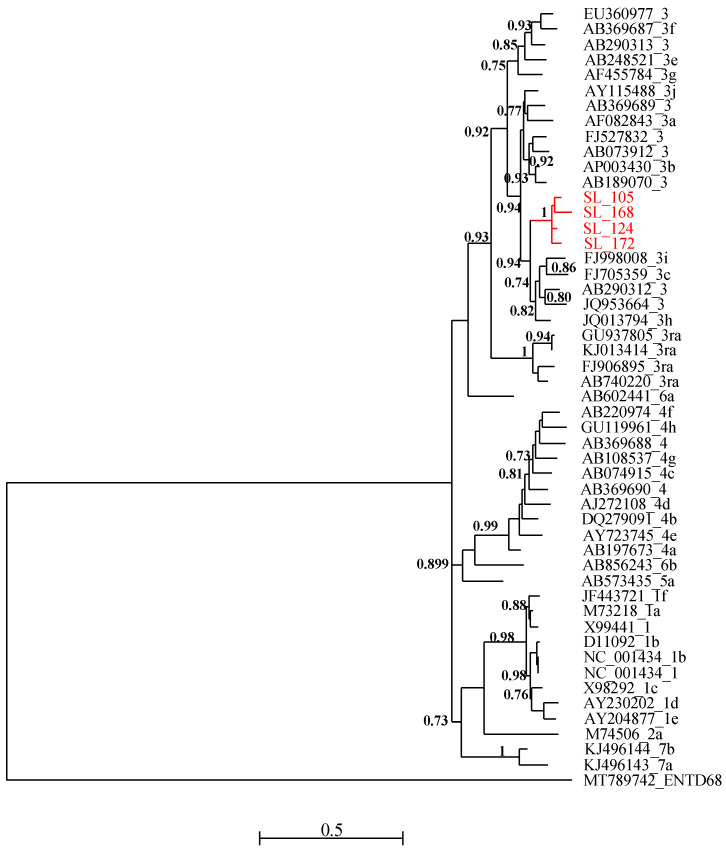
Maximum likelihood (ML) tree [based on the genomic region 5895–6382 relative to NC_001434 reference sequence] showing the relationships between newly obtained HEV partial sequence (ORF-2) isolates from pigs in Senegal (red font) and reference sequences (black font). The tree was constructed using 47 isolates (with tags: Accessions number_genotypes) under the GTR nucleotide substitution model with four Gamma categories using PhyML [66] on SEAVIEW software v5.0.4 [65]. SH-like branches support >0.70 are indicated.

All four RT-PCR-positive samples were successfully sequenced and analyzed with HEV reference sequences. Phylogenetic relationships showed that these new viral strains belonged to genotype 3 and clustered together with maximum aLRT value = 1 (Figure 2). The new HEV sequences have been deposited in Genbank and are available under the following accession numbers: PP483117, PP483118, PP483119, and PP483120.

## 4. Discussion

This study aimed to assess the presence of HEV in pork meat and pork liver samples sold in retail in Saint-Louis, Senegal. This is the first report stating the detection of HEV RNA in meat products from domestic fauna intended for human consumption in Senegal. The results reveal the presence of HEV-RNA in 5.4%; 95% CI [1.7–14%] of pork products (meat and liver samples) collected in the Saint-Louis region. This rate is high compared to what was reported by Modiyinji et al. in their review paper. The authors found a specific prevalence of 3.5% in pigs, while the overall prevalence of HEV-RNA observed in animals (*n* = 6983, including 13 species) was 1.8% in Africa [59]. According to sample types, the highest prevalence was obtained from pig liver samples (22.2%) vs. 3.1% in pig meat samples. This observation is not surprising since the liver is known to be the target organ and the site of HEV replication [43].

Although no statistically significant association was found between HEV-RNA positivity and type of sample, several studies have shown a higher prevalence of HEV in pig liver samples compared to other foods matrices [35,46,50,51,70,71,72]. This confirms the existence of a zoonotic reservoir of HEV in the pig population. According to data from the dedicated literature, many human HEV cases in industrialized countries are related to the consumption of so-called “high-risk” products, i.e., pork products consumed raw or not well-cooked and containing a high proportion of pork liver [71]. Consumers should be concerned that pig liver can be contaminated with HEV and carry a risk of infection if it is not well-cooked, and this emphasizes that pig liver and pork products must be controlled before entering the chain of food [42]. This would limit the spread of HEV as a foodborne pathogen. In this study, the overall prevalence of HEV RNA in pork meat samples collected (3.1%) is high when compared to several other studies screening the same matrix [35,50,72]. However, Di Bartolo et al. reported a similar rate that was found in the present study, i.e., 3% of HEV-RNA positive samples from the Czech Republic out of 112 pork meat sampled at slaughterhouses from the Czech Republic, Italy, and Spain [73]. However, the authors suggested that the presence of the HEV genome was probably due to cross-contamination during slaughtering rather than real virus replication in muscle. The presence of HEV-RNA in pork products probably reflects endogenous HEV particles in infected liver and/or viremic blood [74]. This suggests that there is less risk of contamination when eating pork meat.

In view of these results, our study reveals a possible circulation of HEV in domestic fauna in Saint-Louis. In addition, our previous study conducted among pregnant women showed an overall high HEV seroprevalence of 7.8% in Senegal, with the specific HEV-IgG and HEV-IgM rates of 10.5% and 0.5%, respectively, in Saint-Louis [10]. Therefore, our findings support the need for HEV surveillance using a One Health approach, taking into account the environment and human and animal compartments. Considering HEV as a model of zoonotic pathogens affecting these different compartments, more in-depth and integrated investigations will be relevant to mitigate the associated risks of emergence. It is therefore essential to monitor the health of domestic fauna in order to guarantee the health of human beings. These animals are known as reservoirs, i.e., asymptomatic carriers of HEV. Given that these pigs cohabit with humans in the same environment or may even be consumed by humans, it is important to be aware of certain risk factors to avoid the emergence and re-emergence of certain diseases.

Phylogenetic analysis of the isolated sequences pointed out an exclusive presence of genotype 3. Similar results have been reported in Cameroon [75], Ghana [76], and Burkina Faso [77]. Domestic pigs (*Sus scrofa domesticus*) represent the most important reservoir of the zoonotic genotypes HEV-3 and HEV-4 [78], and several studies have also reported the global distribution of genotype 3 [79]. In many cases, the identification of these genotypes was also made on ORF2 sequences [75], but in shorter sizes. In Senegal, as in neighboring countries, these are the first data describing the presence of HEV in food matrices, particularly meat products. HEV genetic diversity data previously known was limited to genotype 2b found in humans [21].

Given that samples were collected according to their availability in Saint-Louis, the main limitation of this study remains the sample size, especially the pork liver samples. In addition, we were limited by the number of collection sites due to the fact that there are no public pork slaughterhouses in Saint-Louis and that pork is slaughtered and sold in detail in clandestine houses or bars without any regulation or control. These places are known through a very limited network and are little known by the population as a whole, which is the reason why it is rare to find pork sold in retail in Saint-Louis. This could be explained by the fact that the majority of the Senegalese are Muslims, a context in which pig farming is poorly regarded, especially as the consumption of pork in Senegal represents 15% of white meat and is mainly consumed by non-Muslims (representing less than 4% of the population) and expatriates [80,81].

Another limitation of this study was that the ML tree was drawn based on the 485-nt region, which was not sufficient for phylogeny reconstruction using 51 representative isolates. Thus, the characterization of the complete genomes of the isolated strains could contribute substantially to documenting the HEV genotypes circulating in Senegal. Furthermore, this pioneering study on HEV carried out on food products would have made it possible to isolate the first HEV-3 sequences of swine origin in Senegal.

## 5. Conclusions

Our results suggest the potential circulation of HEV in domestic fauna with a contamination rate of 5.4% for pork meat and liver sold in Saint-Louis, Senegal. Therefore, consumption and handling of raw or undercooked contaminated pork meat or pork liver can lead to sporadic cases of HEV infection. On the other hand, the high level of contamination (22.2%) in the pork liver samples highlights a major risk associated with their consumption. Hence, there is a need to inform local communities of the potential circulation of HEV in domestic wildlife. Taking into account these new data, strategies to raise awareness and prevent HEV infection and its spread across the country will have to be implemented. Our results stress the need for a surveillance system that extends this investigation to a national scale while increasing the number of sampling sites and analyzed products. This will ensure continuous monitoring and control of the quality of pork products from farm to fork. A routine HEV surveillance program should be implemented in domestic fauna and among the general population to better know its genetic diversity and circulation in Senegal. Definitively, assessing the activity of HEV strains isolated from pigs by cell culture is necessary. This would enable us to study the association between virus infectivity and the presence of HEV RNA in pork samples.

## Figures and Tables

**Table 1:** Distribution of samples and positivity rate of HEV-RNA in pork sold in Saint-Louis.

Sites	Sample Type		Sample Sizes	Prevalence of HEV (*n*)	95% CI	*p*
	Pork Meat Samples	Pork Liver Samples				
Ndioloffène	29	9	38	10.50% (*n* = 4)	[3.4–25.7%]	
Richard-Toll	17	0	17	0% (*n* = 0)	[0–20.9%]	0.1698
Darou	19	0	19	0% (*n* = 0)	[0–22.9%]	
TOTAL	65	9	74	5.4% (*n* = 4)	[1.7–14%]	

**Table 2:** Positivity rate for HEV RNA in pork (meat and liver) sold in Saint-Louis.

Sample	Sample Sizes	Positive Samples	Prevalence of HEV	95% CI	*p*
Pork meat	65	2	3.1%	[0.53–11.6%]	0.0699
Pork liver	9	2	22.2%	[3.9–59.8%]	

## Data Availability

The data presented in this study are available on request from the corresponding author.
